# Single-cell RNA sequencing reveals transcriptional changes in circulating immune cells from patients with severe asthma induced by biologics

**DOI:** 10.1038/s12276-024-01368-y

**Published:** 2024-12-13

**Authors:** Kyungtaek Park, Ji-Hyang Lee, Eunsoon Shin, Hye Yoon Jang, Woo-Jung Song, Hyouk-Soo Kwon, Yoo Sook Cho, Jong Eun Lee, Ian Adcock, Kian Fan Chung, Jeong Seok Lee, Sungho Won, Tae-Bum Kim

**Affiliations:** 1 Institute of Health and Environment, Seoul National University, 1 Kwanak-ro, Kwanak-gu, Seoul, 151-742 Korea; 2 Department of Allergy and Clinical Immunology, Asan Medical Center, University of Ulsan College of Medicine, Seoul, Korea; 3 DNA Link, Inc, Seodaemun-Gu Bugahyeon-Ro 150, Industry Coop Bldg. 2Nd Fl, Seoul, 120-140 Korea; 4 National Heart and Lung Institute, Imperial College London, London, UK; 5 Genome Insight, Inc., San Diego, La Jolla, CA USA; 6 Graduate School of Medical Science and Engineering, Korea Advanced Institute of Science and Technology (KAIST), Daejeon, Korea; 7 Department of Public Health Sciences, Graduate School of Public Health, Seoul National University, 1 Kwanak-ro, Kwanak-gu, Seoul, 151-742 Korea

**Keywords:** Chronic inflammation, Transcriptomics

## Abstract

Patients with severe eosinophilic asthma often require systemic medication, including corticosteroids and anti-type 2 (T2) cytokine biologics, to control the disease. While anti-IL5 and anti-IL4Rα antibodies suppress the effects of IL-4, IL-5 and IL-13, the molecular pathways modified by these biologics that are associated with clinical improvement remain unclear. Therefore, we aimed to describe the effects of T2-targeting biologics on the gene expression of blood immune cells. We conducted single-cell RNA sequencing (scRNA-seq) of peripheral blood mononuclear cells (PBMCs) from eight patients with severe eosinophilic asthma treated with mepolizumab, reslizumab, or dupilumab. PBMCs were obtained before the initiation of biologics and at 1- and 6-month timepoints after the initiation of treatment to elucidate treatment-induced changes. During treatment, the proportions of T cells/natural killer (NK) cells, myeloid cells, and B cells did not change. However, the composition of classical monocytes (CMs) changed: *IL1B*^+^ CMs were reduced, and *S100A*^+^ CMs were increased. The subsets of T cells also changed, and significant downregulation of the NF-κB pathway was observed. The genes related to the NF-κB pathway were suppressed across T/NK, myeloid, and B cells. The transcriptional landscape did not significantly change after the first month of treatment, but marked changes occurred at six-month intervals. In conclusion, regardless of the type of biologics used, suppression of T2-mediated pathways ultimately reduces the expression of genes related to NF-κB signaling in circulating immune cells. Further studies are warranted to identify potential biomarkers related to treatment response and long-term outcomes.

**Clinical trial registration number:** NCT05164939

## Biologics Transform immune cell gene expression in asthma

Severe asthma is a chronic disease where the airways are inflamed, causing breathing issues even with high-dose inhaled corticosteroids. Current treatments, including biologics, help but don’t fully control the disease. Researchers studied how biologics affect immune cells in severe asthma patients. In this study, they analyzed blood samples from eight patients treated with biologics like mepolizumab, reslizumab, and dupilumab. They used single-cell RNA sequencingto examine changes in gene expression in immune cells over six months. The researchers found that biologics reduced inflammation by altering specific immune cell pathways. They observed a decrease in pro-inflammatory genes and an incrḍease in genes related to immune regulation. These changes suggest that biologics help control severe asthma by modifying immune cell behavior. This summary was initially drafted using artificial intelligence, then revised and fact-checked by the author.

## Introduction

Severe asthma is a chronic inflammatory disease of the airways characterized by uncontrolled symptoms despite the use of high-dose inhaled corticosteroids^1^. For patients with severe asthma, systemic medication, including systemic corticosteroids and additional biologics, is often required to control airway inflammation^2^. Biologics that target type 2 (T2) cytokines such as interleukin (IL)-4, IL-5, and IL-13 have widened the treatment options for patients with severe eosinophilic asthma^3^. Although the inhibition of key T2 cytokines ultimately alleviates eosinophilic inflammation in the airways and leads to clinical improvement^4^, the complex interactive molecular networks modulated by biologic therapy remain largely unknown^5^. In addition, biomarkers that predict treatment response to biologics are lacking, except for the blood eosinophil count, which has satisfactory predictivity^6,7^ only, as treatment response does not always correlate with blood eosinophil levels^8,9^.

Recent advances in omics technology have enabled researchers to further elucidate the pathophysiology of asthma and identify its molecular phenotypes^10,11^. In particular, transcriptomic analysis can provide information on the patterns and dynamics of gene expression related to the severity or the level of control of asthma^12^. More recently, the application of single-cell RNA sequencing (scRNA-seq) has revealed gene expression at the single-cell level in bronchial biopsies of a small number of patients with mild asthma^13^. These scRNA-seq studies have enabled the identification of novel cell types and gene networks that may be involved in the pathogenesis of asthma^14^. To date, no study has investigated the cell-specific transcriptional changes induced by treatment with biologics. Considering that biologic therapies influence circulating blood cells, the identification of alterations in specific pathways involving specific circulating immune cells could provide important insights into the mechanisms of action of these biologic therapies and potential blood biomarkers related to treatment response^3^.

The prospective observational study precision medicine intervention in severe asthma (PRISM)^15^, which includes patients with severe asthma, aims to identify molecular pathways related to the progression of severe asthma and the response to biologics by performing multiomics analyses of serially obtained samples. We hypothesized that scRNA-seq analysis of peripheral blood cells from patients within the PRISM study may reveal novel cell-specific pathways modulated by anti-asthma biologics in addition to the expected T2 pathways. Therefore, we performed scRNA-seq analysis on the peripheral blood immune cells of patients with severe asthma who were successfully treated with the anti-IL-5 biologics mepolizumab and reslizumab and the anti-IL4Ra antibody dupilumab.

## Methods

**Fig. 1:**
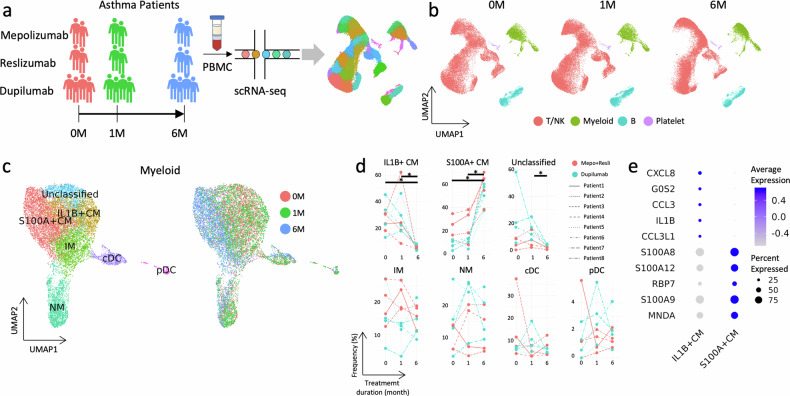
Changes in the transcriptional profiles of blood immune cells at least one month after treatment with biologics. **a** Experimental design illustrating the sample preparation for the study. **b** UMAP plots of all cells classified into T/NK cells, myeloid cells, B cells, or platelets and separated according to treatment duration. **c** UMAP plots showing the subtypes of myeloid cells (left panel) and their distributions according to treatment duration (right panel). **d** Line plots displaying the frequencies of myeloid cell subtypes, where the *x*-axis represents the treatment duration and the *y*-axis represents the frequency. Each line type represents a patient, and the colors indicate the biologics with which they were treated: mepolizumab/reslizumab or dupilumab. Asterisks denote significant differences between the two treatment durations with an FDR of 0.01. **e** Dot plots showing the top 5 highly expressed genes in IL-1B^+^ CMs (first five) and S100A^+^ CMs (last five) compared with other myeloid cell subtypes.

A schematic of the scRNA-seq testing that was performed on peripheral blood mononuclear cells (PBMCs) from eight patients with severe asthma is shown in Fig. 1a. The clinical data and blood samples were obtained consecutively: before initiating treatment with biologics (0 M) and one month (1 M) and six months (6 M) after treatment. The Institutional Review Board of Asan Medical Center approved this study, and all patients provided written informed consent (IRB No. 2019–1676).

### Sample preparation and sequencing

PBMCs were isolated from whole blood using Ficoll‒Paque™ (Miltenyi Biotec, CA). Single-cell RNA library preparation and sequencing were performed by DNA Link, Inc. (Seoul, Korea), the details of which are described in the Online Supplement.

### Data preprocessing

The FASTQ files from all 24 samples from 8 patients were aligned and aggregated using 10x Genomics Cell Ranger 6.0.0^16^. The raw count matrix consisted of 36,601 features and 102,951 cells. Features detected in fewer than 3 cells and cells with fewer than 200 non-zero expressed features were removed. Next, the number of detected features and their counts on a log10 scale and the ratio of mitochondrial genes were calculated for each cell. Cells that did not deviate more than 5 median absolute deviations (MADs) from the medians of the number of detected features and their counts and 10 MADs from the median of the ratio of mitochondrial genes were retained. Among the remaining cells, doublet cells with hybrid scores greater than 0.8 were removed using the scds R package (v1.0.0)^17^. Finally, 25,242 features of 89,728 cells were used for the analysis. The data were normalized via the *NormalizeData* function with its default options in Seurat (v4.0.5)^18^. Throughout the entire process, the Seurat (v4.0.5) and ggplot2 (v3.4.0)^19^ packages within R (v4.1.1) were used.

### Cell type annotation

The cell types were manually annotated using specific markers (Supplementary Fig. 1a). These cell types and CD4^+^ T cells were clustered into their subtypes according to established markers (Supplementary Fig. 1b–d and Supplementary Fig. 2). CD4^+^ T cells were further classified into State 0 (S0) and State 1 (S1) groups on the basis of clusters, with State 0 consisting primarily of cells from cluster 0 M and 1 M and State 1 consisting primarily of cells from cluster 6 M. To visualize the cells on a two-dimensional plot, the principal components (PCs) of the gene expression matrix were calculated based on 2,000 highly variable genes via the *RunPCA* and *FindVariableFeatures* functions using their default options in Seurat. We selected the optimal number of PCs for clustering the cells based on elbow plots, which represent the standard deviation of each PC. Next, with the optimal number, UMAP scores were calculated via the *RunUMAP* function in Seurat. For further clustering, the *FindNeighbors* and *FindClusters* functions in Seurat were used.

### Association test between frequencies of cell types and duration of treatment

The frequencies of cell types were calculated by dividing the number of each cell type by the total number of cells obtained from a single patient. To determine whether the frequencies of the cell types were associated with the duration of treatment, linear mixed effect models were used. Patients were considered random effects in these models. The full model included the status variable, whereas the reduced model only consisted of the intercept term as an explanatory variable. The significance of the status variable was estimated using the difference in likelihoods of the two models, and *p* values were adjusted via the Benjamini‒Hochberg method.

### Differentially expressed gene (DEG) analysis

The *FindMarkers* function in Seurat was used to compare the gene expression levels between groups. For this test, we used MAST and used the other settings at their default values. Genes whose false discovery rate (FDR) was less than 0.01 and whose log2-fold change was greater than 0.25 compared with the other group(s) were considered differentially expressed genes (DEGs). To test the effects of treatment duration on gene expression levels, T/natural killer (NK), myeloid, and B cells at 0 M and 1 M were combined into one group and compared with their counterparts at 6 M, while CD4^+^ T cells in the S0 and S1 groups were compared with each other. When comparing subtypes, each subtype was compared with the other subtypes, and when two groups were designated, only those subtypes were compared. Dot plots and heatmaps were drawn using the *DotPlot* and *DoHeatmap* functions in Seurat, respectively. The DEGs were further analyzed to identify pathways or transcription factors (TFs) associated with these genes via MSigDB Hallmark 2020 and TRRUST Transcription Factors 2019 implemented in Enrich R (https://maayanlab.cloud/Enrichr/)^20^. The top 5 most significant terms associated with DEGs at 0 M and 1 M or with S0 were extracted from each analysis, and their *p* values were compared with those of the association test between the terms and DEGs at 6 M and for S1 cells.

### Cell‒cell communication analysis

Cell-cell communication analyses were conducted via the CellChat R package (v1.5.0)^21^ for CD4^+^ T-cell subtypes (excluding T-cell receptor (TCR)-stimulated subtypes), *IL1B*^*+*^ and *S100A*^*+*^ classical monocytes, and naïve and memory B cells. Ligand‒receptor interaction data were obtained from CellChatDB.human, which was provided by the package mentioned above. Signaling pathways in the inferred cellular communication networks were identified on the basis of biologics and treatment duration. Chord diagrams of signaling pathway networks were drawn via the *netVisual_aggregate* function in the package with its default options. The difference in the number of interactions between S1 and S0 is shown in raster plots.

## Results

### Characteristics of the study participants

A total of eight patients with severe eosinophilic asthma were included in the analysis. The baseline data and changes in clinical variables, including inflammatory markers, lung function, asthma control test score, number of acute exacerbations, and use of maintenance oral corticosteroids (OCS) over time, are described in Table 1 and Supplementary Table 1. Two patients were treated with mepolizumab (100 mg IV every 4 weeks), two patients were treated with reslizumab (3 mg/kg IV every 4 weeks), and four patients were treated with dupilumab (600 mg loading dose followed by 300 mg IV every 2 weeks) for up to six months. Four participants treated with anti-IL-5 antibodies had a significantly improved FEV1 (% predicted) (53.00 ± 6.48 vs. 72.75 ± 10.21, *p* = 0.008) (Table 1) along with a reduced blood eosinophil count (1244 ± 1088.4 vs. 36.5 ± 13.99, *p* = 0.030) after 6 months of biologic therapy (Supplementary Table 1). Two of the four patients treated with dupilumab succeeded in the withdrawal of maintenance OCS (Table 1). In all the molecular analyses, the results of the patients on mepolizumab and reslizumab were combined and compared with the results of the patients on dupilumab.

**Table 1:** Characteristics of patients with severe asthma and the effects of biologic therapy.

No.	Age/sex	Drug	FEV1 (pred %)			ACT score			Blood eosinophils (cells/µL)			Number of exacerbations		OCS maintenance	
			0 M	1 M	6 M	0 M	1 M	6 M	0 M	1 M	6 M	Prev.6 months	During treatment	Before treatment	During treatment
1	43/F	Mepolizumab	46	60	60	11	12	20	2856	103	30	9	1	No	N/A
2	74/F	Mepolizumab	58	75	73	25	25	25	682	70	50	0	0	No	N/A
3	50/F	Reslizumab	59	85	85	18	10	24	509	50	46	0	0	No	N/A
4	62/F	Reslizumab	49	46	73	19	19	21	929	166	20	5	0	No	N/A
5	74/F	Dupilumab	53	59	53	20	20	24	482	400	284	0	0	No	N/A
6	58/M	Dupilumab	65	87	86	16	21	25	312	1794	252	13	0	Yes	Withdrawal
7	62/M	Dupilumab	30	37	33	18	21	20	283	598	552	0	1	Yes	No withdrawal
8	46/M	Dupilumab	73	99	90	13	22	24	202	2232	1782	1	0	Yes	Withdrawal

### Transcriptional profiles of blood immune cells after biologic treatment

From the 24 samples from 8 patients analyzed over 3 timepoints, we identified 25,242 features and 89,728 cells. Marker genes were used to call immune cell types (Supplementary Fig. 1a), including the subtypes of T/NK cells (Supplementary Fig. 1b), myeloid cells (Supplementary Fig. 1c), and B cells (Supplementary Fig. 1d). A UMAP projection of T/NK cells, myeloid cells, B cells, and platelets from the combined data plotted according to the treatment duration revealed that the distribution of cells at 6 M was different from that at 0 M and 1 M (Fig. 1b). However, the overall proportions of T/NK, myeloid, and B cells remained similar during treatment with biologics (Supplementary Fig. 1e). Several clusters belonging to these cell types were differentially distributed at 6 M compared with 0 M and 1 M (Fig. 1c and Supplementary Fig. 1f). These findings suggest that the states of cell subtypes are altered by treatment with biologics. A subanalysis of myeloid cells revealed the presence of classical monocytes (CMs), intermediate monocytes (IMs), nonclassical monocytes (NMs), unclassified monocytes, conventional dendritic cells (cDCs), and plasmacytoid dendritic cells (pDCs). CMs were further divided into *S100A*^+^ and *IL1B*^*+*^ CMs (Fig. 1c). The predominant changes in myeloid cells at 6 M occurred mainly among CMs (Fig. 1d), while the *IL1B*^+^ CMs that were dominant at 0 M and 1 M were supplanted by *S100A*^+^ CMs at 6 M irrespective of the type of biologic therapy. The most highly expressed DEGs in *IL1B*^+^ CMs were *IL1B*, *CXCL8*, and *CCL3*, whereas *S100A*^+^ CMs were characterized by high expression of S100 protein family genes, including *S100A8*, *S100A9*, and *S100A12* (Fig. 1e).

The transcriptional profiles of T/NK cells were well separated between the 1 M and 6 M treatment groups within each subtype of T/NK cells (Supplementary Fig. 1f); however, the proportion of each subtype did not change significantly over time (Supplementary Fig. 1g). In contrast, biologic therapy failed to induce any significant changes in B-cell clusters or in the proportions of subtypes present in the blood (Supplementary Fig. 1h and 1i).

### Transcriptomic profile of blood CD4^+^ T cells after biologic treatment

**Fig. 2:**
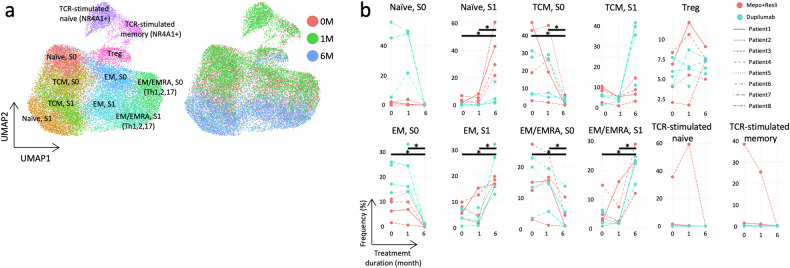
Altered subsets of CD4^+^ T cells in the blood after six months of treatment with biologics. **a** UMAP plots showing the subtypes of CD4^+^ T cells (left panel) and their distributions according to treatment duration (right panel). **b** Line plots displaying the subtypes, where the *x*-axis and *y*-axis represent the treatment duration and frequency, respectively. The line types and colors correspond to the conditions, and asterisks indicate significant changes between the treatment durations with an FDR of 0.01. S0 and S1 represent States 0 and 1, respectively.

We expected the profile of CD4^+^ T cells, which drive type 2 inflammation in asthma, to be modified with anti-T2 biologics. As indicated above (Supplementary Fig. 1f), the transcription profile of CD4^+^ T cells at 6 M was considerably different from that at 0 M and 1 M. Therefore, we classified these T cells not only into classical subtypes but also into State 0 (S0) and State 1 (S1) groups, which represent different types or states of these cells. Thus, the cells were annotated as one of the following: naïve S0 and S1, T central memory (TCM) S0 and S1, effector memory (EM) S0 and S1, and terminally differentiated effector memory cells (EM/EMRA) S0 and S1. Different states or cell types were not observed for regulatory T cells (Tregs) or TCR-stimulated naïve or memory cells after 6 M; thus, these cells did not transition into different states (Fig. 2a). Naïve S0, TCM S1, Treg, TCR-stimulated naïve, and TCR-stimulated memory cells did not significantly change after 6 months. In contrast, the enrichment of naïve S1, EM S1, and EM/EMRA S1 cells was significantly increased at 6 M, irrespective of the treatment received. The enrichment of TCM S0, EM S0, and EM/EMRA S0 cells was significantly decreased at 6 M across all treatments (Fig. 2b).

### Genes involved in the NF-κB pathway are downregulated across cell types

**Fig. 3:**
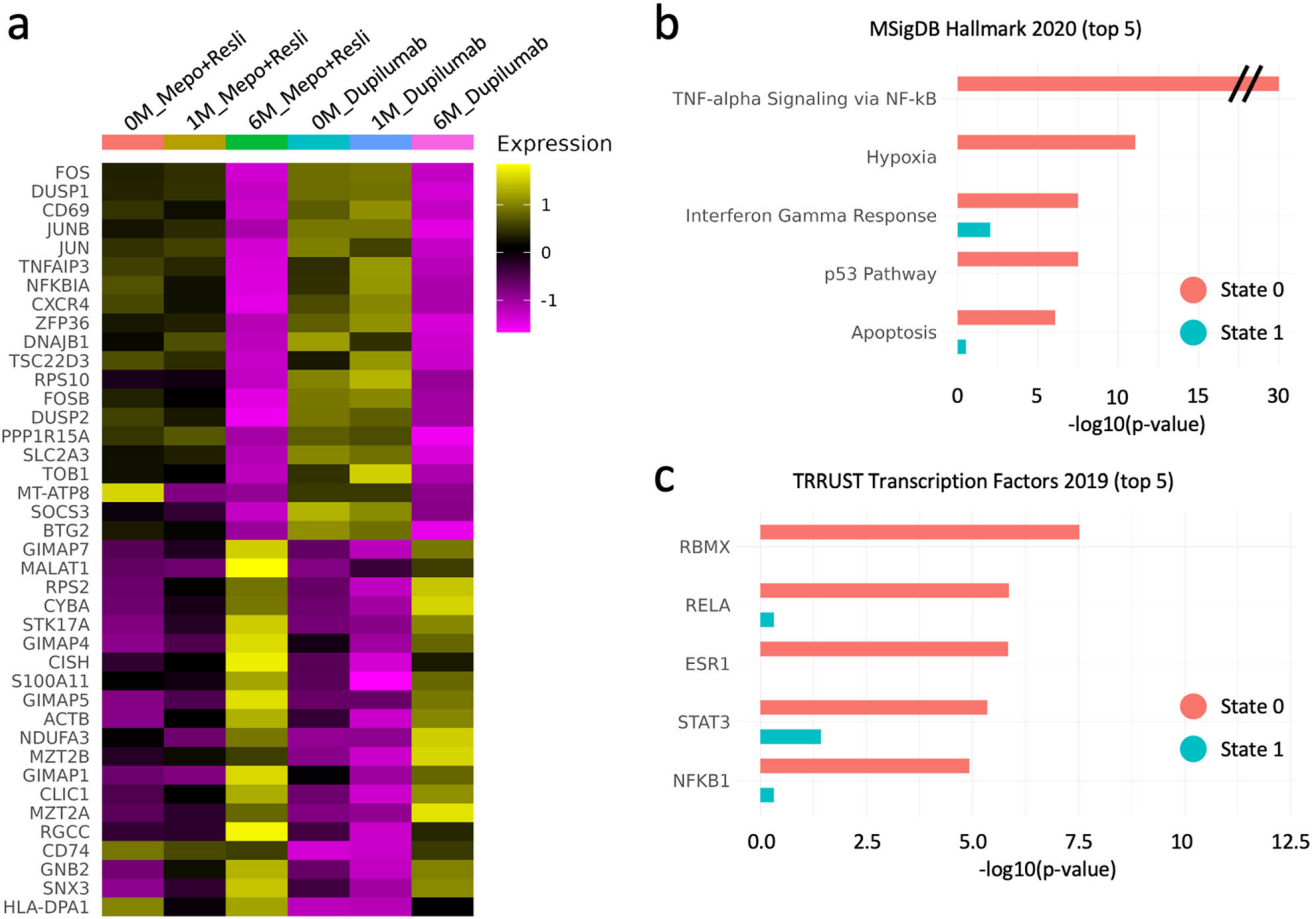
Downregulation of genes involved in the NF-κB pathway across all cell types. **a** Heatmap displaying the expression levels of DEGs that were significantly different between S0 and S1 CD4^+^ T cells and categorized by treatment duration and biologic conditions. The top 20 downregulated and upregulated DEGs in CD4^+^ T S1 cells compared with S0 cells are shown on the *y*-axis. **b** Top 5 MSigDB hallmark 2020 pathways and their *p* values associated with upregulated DEGs in State 0. The *y*-axis represents the names of the pathways, and the *x*-axis represents the −log10 of their *p* values. Each pathway has two bars representing *p* values calculated for upregulated DEGs in S0 (red) and S1 (turquoise). **c** The top 5 TRRUST transcription factors and their *p* values associated with upregulated DEGs in S0 (red). The turquoise bars represent *p* values of the corresponding pathways associated with upregulated DEGs in State 1.

When we compared the gene expression levels between S0 and S1 CD4^+^ T cells, activator protein 1 (AP-1) genes such as *FOS*, *FOSB*, *JUN*, and *JUNB* were significantly downregulated, whereas GIMAP family genes (*GIMAP1*, *GIMAP4*, *GIMAP5*, and *GIMAP7*) were significantly upregulated in the S1 group (Fig. 3a). Pathway and TF analyses of the DEGs between the two groups revealed that the downregulated DEGs in the S1 group were associated with TNF-α signaling via NF-κB, followed by hypoxia, interferon (IFN)-γ responses, the p53 pathway, and apoptosis (Fig. 3b). Among the top 20 downregulated DEGs, 14 genes, including AP-1 genes, *DUSP1/2*, *CD69*, *TNFAIP3*, *NFKBIA*, *ZFP36*, *PPP1R15A*, *SLC2A4*, *SOCS3*, and *BTG2*, were involved in the NF-κB pathway. In the TF analysis, the two main subunits of NF-κB, RELA and NFKB1, were significantly associated with the downregulated DEGs in the S1 group (Fig. 3c). In addition, STAT3, a target of IL-4/5/13, was significantly associated with these DEGs, with an FDR of 0.05.

When the gene expression levels in individual T/NK, myeloid, and B-cell subsets were investigated, a similar pattern was observed. Among the top 20 downregulated DEGs, NF-κB pathway-related genes, such as *FOS*, *FOSB*, *JUN*, *DUSP1*, *NFKBIA*, *ZFP36*, and *PPP1R15A*, were shared among the three main cell types, namely, T/NK cells, myeloid cells, and B cells (Supplementary Fig. 3a). TNF-α signaling via NF-κB was the most significant pathway of the cell types, followed by the hypoxia and apoptosis pathways (Supplementary Fig. 3b). In contrast to the reduction in the IFN-γ response and UV response pathways observed in T/NK cells and B cells, myeloid cells exhibited a loss in gene expression related to the inflammatory and p53 pathways. In the TF analyses, RELA and NFKB1 were also commonly found in T/NK, myeloid, and B cells and were among the top 5 associated transcription factors with downregulated DEGs at 6 months (Supplementary Fig. 3c).

### Different effects of biologics on CD4^+^ T cells according to the mechanism of action

**Fig. 4:**
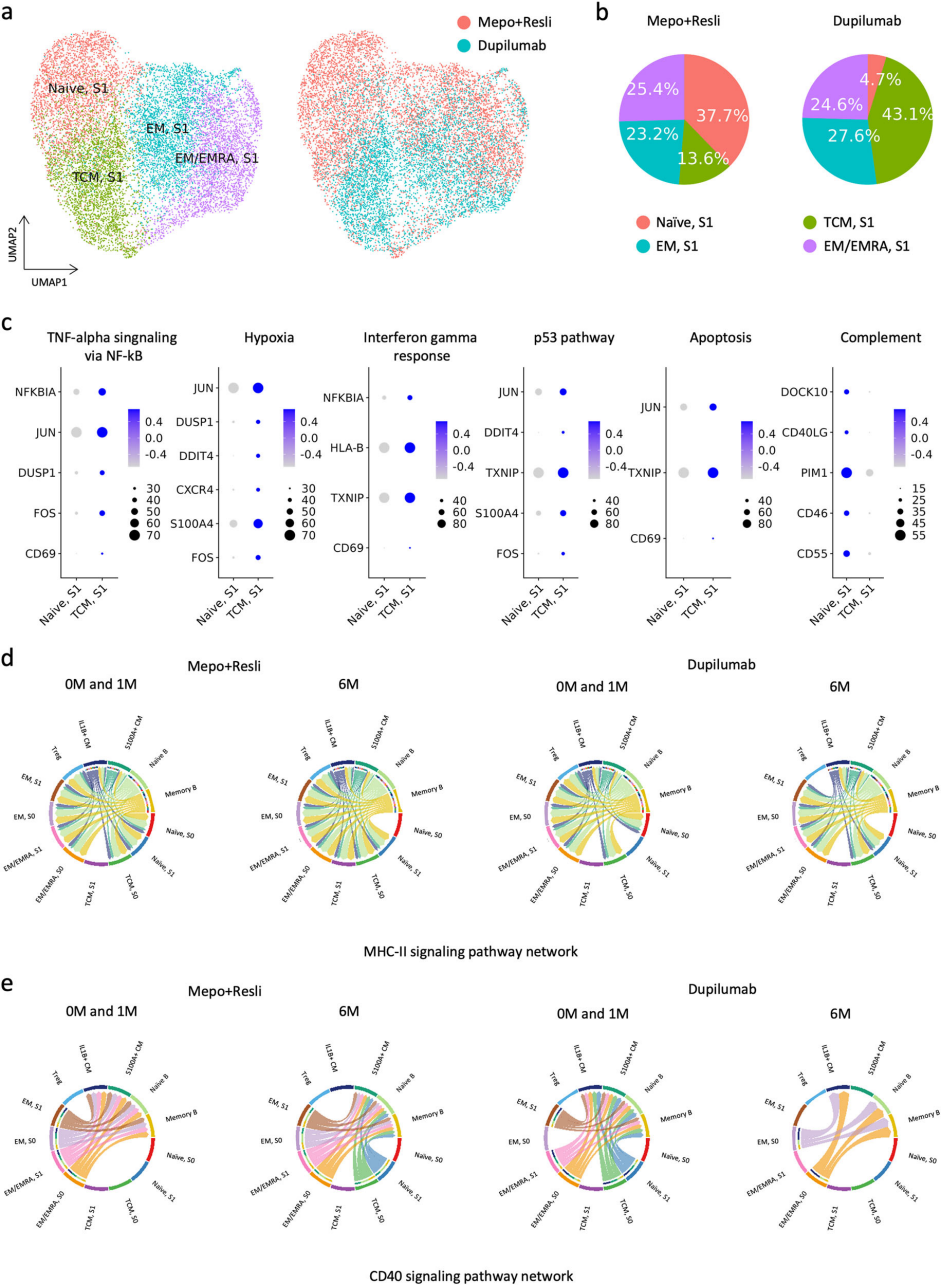
Different effects of biologics on CD4^+^ T cells according to their mechanisms of action. **a** Dimension plots of CD4^+^ T S1 cells plotted according to subtype (left panel) and biologics type (right panel). On the right panel, the cells associated with mepolizumab/reslizumab and dupilumab are colored red and turquoise, respectively. **b** Pie charts show the frequencies of the subtypes according to the biologics type. **c** Dot plots of DEGs included in the MSigDB hallmark 2020 pathways were significantly associated with upregulated DEGs in TCM S1 cells compared with naïve S1 cells (the first five), and vice versa (the last). The colored bars and circles represent the average expression and percent expression of the corresponding genes according to subtype, respectively. **d** Chord plots of the MHC-II signaling pathway network of the 0/1 M and 6 M groups of the mepolizumab/reslizumab and dupilumab groups. **e** Chord plots of the CD40 signaling pathway network of the 0/1 M and 6 M groups in patients in the mepolizumab/reslizumab and dupilumab groups. S0 and S1 represent States 0 and 1, respectively.

Since different compositional changes in CD4^+^ T cells were noted according to the mechanism of action of biologics by 6 M (Fig. 2b), we extracted and separately analyzed S1 CD4^+^ T cells (Fig. 4a). The proportions of the naïve S1 subtype were 37.7% and 4.7% in the mepolizumab/reslizumab and dupilumab groups, respectively, whereas those of the TCM S1 subtype were 13.6% and 43.1% in the mepolizumab/reslizumab and dupilumab groups, respectively (Fig. 4b). Although patients treated with dupilumab had different OCS maintenance statuses (Table 1), the frequencies of naïve and TCM S1 subtypes were not different between patients who underwent withdrawal during treatment (patients 6 and 8) and patients who did not (patient 7) (Fig. 2b and Supplementary Table 2). The downregulated DEGs in the naïve S1 CD4^+^ T-cell subtype compared with those in the TCM S1 CD4^+^ T-cell subtype were significantly associated with TNF-α signaling via NF-κB, hypoxia, the IFN-γ response, the p53 pathway, and apoptosis (Fig. 4c). Moreover, the downregulated DEGs in the TCM S1 CD4^+^ T-cell subtype compared with those of the naïve S1 CD4^+^ T-cell subtype were significantly associated with the complement pathway (Fig. 4c).

After treatment, the number of cell‒cell interactions generally decreased; specifically, the number of interactions between CMs or B cells and naïve S0 CD4^+^ T cells decreased the most across all treatments (Supplementary Fig. 4a). However, distinct differences were observed between the results after mepolizumab/reslizumab and those after dupilumab treatment, as the former resulted in enhanced connections in TCM S1 cells and the latter resulted in enhanced connectivity within naïve S1 T cells (Supplementary Fig. 4a). Connectivity maps of all the studied cell types revealed differences in S0 and S1 with respect to the MHC-II signaling pathway network (Supplementary Fig. 4b) but not in the CD40 signaling pathway network across all treatments (Supplementary Fig. 4c). Separation of the two treatment groups revealed a greater impact of dupilumab than of mepolizumab/reslizumab on both the MHC-II signaling pathway network (Fig. 4d) and the CD40 signaling pathway (Fig. 4e). The interactions between CD4^+^ T and B cells considerably decreased only in patients treated with dupilumab (Fig. 4d, e).

## Discussion

In the present study, we delineated the alterations in the cellular transcriptomic profiles of specific PBMC subsets from patients with severe eosinophilic asthma treated with mepolizumab, reslizumab, or dupilumab for six months. T2-targeting biologics reduced *IL1B*^+^ CMs and increased *S100A*^+^ CMs. These biologics also altered the composition of subsets of CD4^+^ T cells, which indicates significant downregulation of the NF-κB pathway across immune cells. Key regulatory pathways, including MHC-II and CD40 signaling and links to B-cell activation, were differentially affected in the dupilumab- and mepolizumab/reslizumab-treated patient groups. Transcriptional changes in PBMCs occurred at least one month after the initiation of treatment with biologics, which reflects altered systemic conditions induced by biologics.

Previous studies utilizing scRNA-seq analyses of immune and structural cells in humans and mice have contributed to our understanding of the pathophysiology of asthma^14^. In terms of human studies, Chen and colleagues compared the transcriptional characteristics of PBMCs from patients with severe asthma and those from healthy controls^22^. Although the composition of the cell subsets was similar between the groups, increased expression of *JAK1*, the long noncoding RNA *NEAT1*, and *IL32* was noted in specific cell types of patients with severe asthma. Li and colleagues reported the results of single-cell transcriptomics of immune cells in bronchoalveolar lavage fluid during asthma exacerbation, which highlighted an increase in CD8+ T cells, monocytes, and macrophages and the distinct expression of proinflammatory cytokine genes and transcription factors associated with asthma exacerbation^23^. However, longitudinal serial data from scRNA-seq of immune cells from patients with asthma over time and following therapy are lacking^14^.

By alleviating eosinophilic inflammation, biologics that target key T2 cytokines have shown therapeutic benefits in severe eosinophilic asthma^3^. Several studies have evaluated the impact of biologics on circulating immune cells other than granulocytes in patients with asthma. In 2003, Buttner and colleagues reported that treatment with mepolizumab did not alter the distributions of T-cell subsets, B cells, natural killer cells, memory cells, naïve cells, or γδT cells^24^. Another study revealed that mepolizumab reduced the proportions of CD45^+^, CD8^+^ and NKT-like cells in the blood and increased the Treg population^25^. Lommatzsch and colleagues reported an increase in immature B cells after six months of treatment with dupilumab but not with benralizumab^26^. However, the clinical relevance of changes in the proportions of specific cell types remains uncertain. Our analysis revealed that treatment with biologics induced changes in subsets of CMs and CD4^+^ T cells, which suggests the importance of these cell types in the pathogenesis of severe asthma. A greater proportion of blood CMs has been reported in patients with severe asthma than in those with nonsevere asthma^27^, and CD4^+^ T cells are known to play an essential role in the development and progression of asthma^28^. Among CMs, the proportion of the *IL1B*^+^ subgroup was reduced, whereas that of the *S100A*^+^ subgroup was increased. The finding of decreased *IL1B* transcripts is consistent with suppression of the NF-κB and AP-1 pathways since IL-1 is associated with innate cytokines that activate the inflammatory process^29^. IL-1β is involved in chronic inflammatory diseases, and an association between *IL1B*^*hi*^ monocytes derived from PBMCs and active inflammatory arthritis has been reported^30^. However, the clinical relevance of increased *S100A*^+^ transcripts in CMs is uncertain. S100A proteins act as damage-associated molecular patterns, and elevated gene expression of some isoforms has been suggested as a prognostic marker in various pathologic conditions^31^. Considering that S100A8/9 induces neutrophil activation and has been reported as a marker of several inflammatory diseases, upregulation of *S100A* genes may indicate a shift toward the recovery of T2-skewed inflammation, as reflected by a relative increase in T1 inflammation^32^. The interpretation of increased *S100A*^+^ CMs in severe asthma requires further study with a longer observation period and a larger number of patients.

We further revealed that treatment with biologics induced downregulation of the key proinflammatory NF-κB and AP-1 pathways regardless of the biologic used. NF-κB and AP-1 are key players in severe relative steroid-refractory chronic airway inflammation^33^, and persistent NF-κB activation has been observed in patients with severe uncontrolled asthma^34^. In addition, external stimuli, including allergens, endotoxins, and microbial infections, stimulate activation of the NF-κB pathway. A reduction in NF-κB activity in lung tissue has been observed in patients treated with inhaled corticosteroids^35,36^. Considering that glucocorticoids are potent inhibitors of NF-κB activation, these findings explain the steroid-sparing effect of biologics, including dupilumab, which exerts anti-inflammatory effects despite transient elevation of blood eosinophils^9,37^. The observed downregulation of the NF-κB pathway across multiple PBMC types following treatment with biologics suggests the suppression of overactive immune responses and a potential reduction in immune-mediated tissue damage^33^. The downregulation of STAT3, which is involved in the activation of Th2 and Th17 cells, was also noted in the analysis^38^. In asthma, STAT3 participates in airway remodeling via the activation of M2 macrophages and fibroblasts^39^. The reduction in the main inflammatory signaling pathways, including the NF-κB and JAK/STAT signaling pathways, in PBMCs suggests the resolution of systemic inflammation after treatment with biologics.

Despite their similar efficacy in terms of clinical outcomes, mepolizumab and reslizumab bind to IL-5, whereas dupilumab blocks IL-4Rα^3^. When the composition of CD4^+^ T cells was compared according to the mechanisms of action of these biologics, downregulated DEGs in the TCM CD4^+^ T-cell S1 subtype were found to be related to the complement pathway, which was observed in patients who were treated with anti-IL5 biologics. Components of complement are known to regulate the adaptive immune response by binding to their receptors expressed on T cells^40^, which suggests that treatment with mepolizumab or reslizumab might also inhibit the complement pathway. An increase in sputum C3c, a marker of complement activation, has been reported to be a predictor of a suboptimal response to mepolizumab and reslizumab^8^. Network analysis also revealed different cell‒cell interactions in both the MHC-II and CD40 signaling pathways between biologics that target IL-5 and those that target IL-4/13. These findings indicate heterogeneity in the interplay between immune cells during the overall anti-inflammatory process according to the type of biologic used.

In contrast to changes in the clinical variables that started within four weeks after the initiation of biologics, the transcriptional profiles of immune cells markedly differed between one and six months. This aligns with the rapid change in eosinophils seen with T2-targeted therapies, followed by a slower, systemic anti-inflammatory response involving other immune cells. These findings highlight eosinophils as key effector cells and support the concept of severe asthma as a systemic disease with evolving immune involvement. Although the changes in asthma-related outcomes varied among the participants, they exhibited similar changes in circulating immune cell activation states following biologic therapy. The differences in lung function and asthma control suggest that the presence of additional factors contributes to respiratory symptoms, including immune reactions with structural cells in the airways^3^. Future studies should investigate whether changes in NF-κB, AP-1, and S100A pathway activation in blood cells reflect similar changes in the airways of patients with severe eosinophilic asthma following successful biologic treatment.

This study has several limitations. First, the number of included participants was small. However, longitudinal changes were analyzed for each participant, which minimized the potential nuisance effect caused by heterogeneity among all participants. Second, the participants exhibited good responses to treatment with biologics. Therefore, we could not identify a predictive signature or potential biomarker for treatment response to biologics. To address this, a follow-up study including patients with a broader range of treatment responses is needed. Third, the analyzed blood samples did not include granulocytes, including eosinophils and neutrophils, which could also play an important role in severe asthma. Granulocytes, particularly neutrophils and eosinophils, are highly fragile and prone to lysis during isolation and processing, which can result in the loss of cellular content and poor-quality RNA. Additionally, abundant RNases in these cells lead to rapid RNA degradation, which increases the difficulty of the 10x Genomics analysis^41^. Due to these technical limitations, we prioritized the analysis of PBMCs, which are more stable and provide high-quality RNA for scRNA-seq. Nevertheless, the deconvolution of bulk RNA-seq data from these patients may indicate alterations in the blood granulocyte status with different biologic therapies. Finally, we did not investigate the changes in the structural cells of the airway, which is a target organ in asthma. However, our study revealed alterations in the transcriptomic profiles of circulating immune cells of patients with severe asthma induced by treatment with biologics. The transcriptomic changes evident six months after treatment with biologics highlighted the downregulation of the NF-κB, AP-1, and STAT signaling pathways. Further studies with larger populations and longer treatment durations are warranted to identify potential biomarkers related to treatment response and long-term outcomes.

## Supplementary information

Supplementary materials
