# Supplementary material for: Single-cell RNA sequencing reveals transcriptional changes in circulating immune cells from patients with severe asthma induced by biologics
Source: Exp Mol Med. 2024 Dec 13;56(12):2755–62. doi: 10.1038/s12276-024-01368-y (PMC11671581; doi:10.1038/s12276-024-01368-y)

Online Supplement

Methods

Sample preparation and sequencing

PBMC and granulocyte samples were counted using the Countess™ Automated Cell Counter (Invitrogen, Carlsbad, CA) with trypan blue for evaluating the cell viability. PBMC and granulocyte samples were mixed at a 1:1.2 ratio. Mixed samples were prepared for single-cell RNA sequencing using the Chromium Single-Cell 5′ Reagent version 1.1 kit (PN 1000165) and Chromium Single-Cell Controller (10x Genomics, CA). Cells were subsequently loaded into the Single-Cell G Chip (PN 1000120) for gel bead-in-emulsion (GEM) generation and barcoding, targeting for a cell recovery of 10,000 cells per sample. Reverse transcription, RT clean-up, and cDNA amplification were performed to isolate and amplify cDNA for downstream 5′ gene or enriched V(D)J library construction according to the manufacturer's protocol. Libraries were constructed using the Chromium Single-Cell 5′ reagent kit v1.1 (PN 1000165) and 5′ Library Construction Kit (PN 1000020) according to the manufacturer's protocol. For sequencing, the library concentration was assessed using a Qubit 2.0 Fluorometer and dsDNA HS Assay Kit (Thermo Fisher Scientific, Waltham, MA) according to the manufacturer's protocol. Single-cell 5′ gene expression libraries were sequenced using an Illumina NovaSeq 6000 system following standard protocols for a read length of 2 x 100 bp.

Supplementary Table 1. Effect of biologic therapy on inflammatory markers

No.	Age/ sex	Drug	**FeNO** (ppb)			**Sputum eosinophil** (%)			**Blood eosinophils,** absolute count (proportion)			**Blood neutrophils,** absolute count (proportion)		
			0M	1M	6M	0M	1M	6M	0M	1M	6M	0M	1M	6M
1	43/F	Mepolizumab	.	.	155	76	47	.	2856 (23.8)	103 (1.9)	30 (0.4)	6372 (53.1)	3775 (69.9)	5472 (72.0)
2	74/F	Mepolizumab	49	41	29	90	5	.	682 (12.4)	70 (1.4)	50 (0.9)	2277 (41.4)	2510 (50.2)	2576 (46.0)
3	50/F	Reslizumab	86	75	94	1	0	.	509 (7.6)	50 (0.6)	46 (0.6)	3551 (53)	5354 (64.5)	4736 (61.5)
4	62/F	Reslizumab	57	70	79	47	31	.	929 (12.9)	166 (2.6)	20 (0.3)	4370 (60.7)	3605 (59.1)	4877 (73.9)
5	74/F	Dupilumab	62	20	39	53	53	.	482 (7.3)	400 (7.4)	284 (4.9)	3946 (59.8)	3218 (59.6)	3323 (57.3)
6	58/M	Dupilumab	96	50	24	30	.	.	312 (5.2)	1794 (21.1)	252 (4.0)	3072 (51.2)	3392 (39.9)	3415 (54.2)
7	62/M	Dupilumab	12	.	11	4	.	7	283 (2.5)	598 (4.9)	552 (4.8)	6735 (59.6)	7283 (59.7)	7625 (66.3)
8	46/M	Dupilumab	135	55	24	0	.	0	202 (2.3)	2232 (15.5)	1782 (17.3)	6802 (77.3)	8640 (60)	5583 (54.2)

Supplementary Table 2. Frequencies of S1 $CD4^+$ T subtypes across patients with dupilumab treatment.

No.	Naïve, S1	TCM, S1	EM, S1	EM/EMRA, S1
5	14.3%	19.0%	38.9%	27.8%
6	2.71%	44.0%	23.9%	29.4%
7	4.41%	47.2%	31.1%	17.3%
8	2.32%	49.3%	19.8%	28.6%

Supplementary Fig. 1. Changes in transcriptional profiles of blood immune cells at least one month after treatment with biologics

(a) Dot plots of markers for annotating T/NK cells, myeloid cells, B cells, and platelets. Dot plots of markers for subtypes of (b) T/NK cells, (c) myeloid cells, and (d) B cells. (e) Pie charts representing the frequencies of T/NK cells, myeloid cells, B cells, and platelets according to the treatment duration. (f) UMAP plots and (g) line plots of T/NK cells. (h) UMAP plots and (i) line plots of B cells. (f-i) The UMAP plots show the subtypes (left panel) and their distribution according to the treatment duration (right panel), and the line plots display the frequencies of the subtypes, with the x-axis and y-axis representing the treatment duration and frequency, respectively. Each line type represents a patient, and the colors represent the biologics. They were treated with, mepolizumab/reslizumab or dupilumab. EM/EMRA and gd T cells represent terminally differentiated effector memory and gamma-delta T cells, respectively.

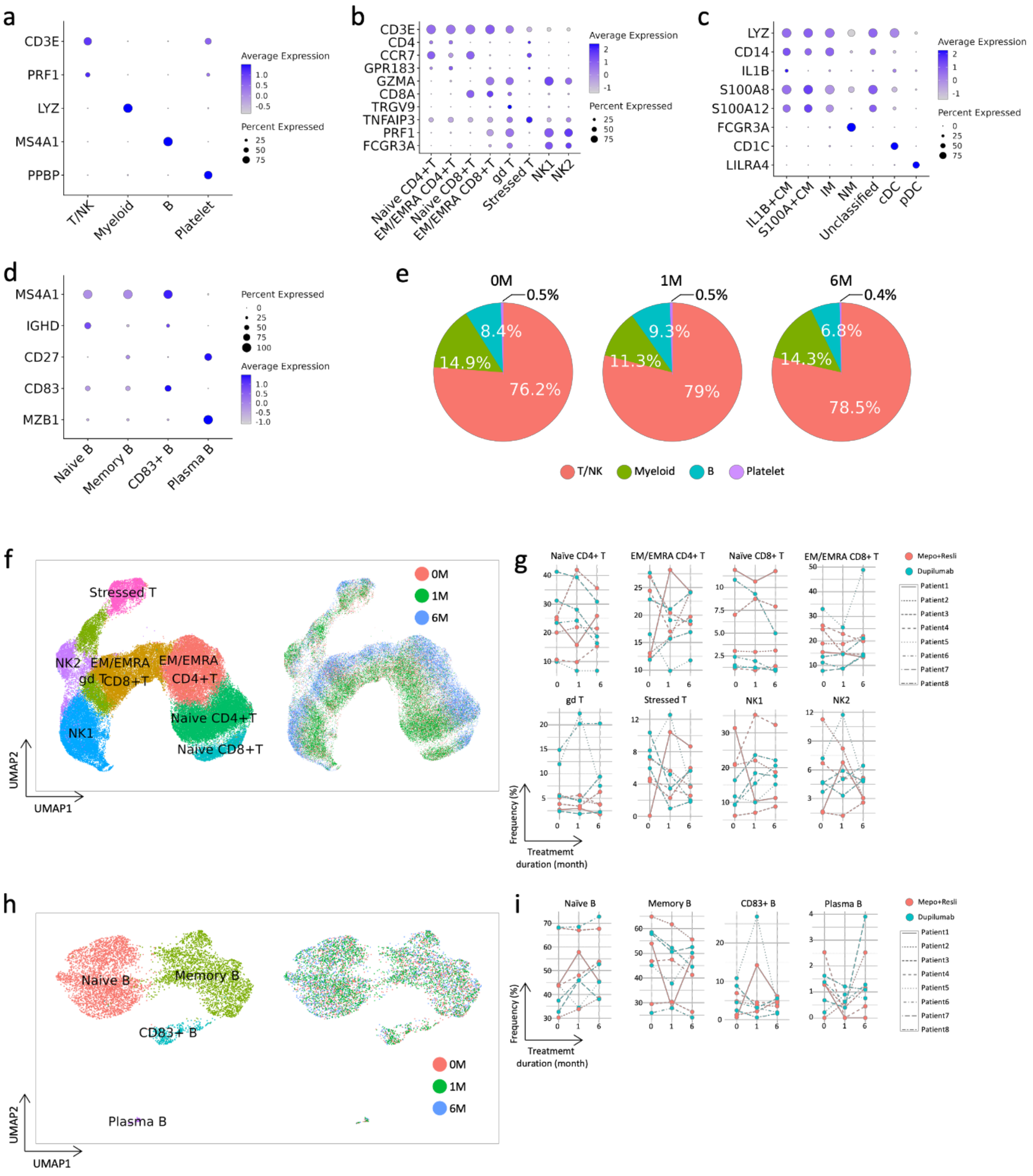
a
CD3E
PRF1
LYZ
MS4A1
PPBP
T/NK
Myeloid
B
Platelet
Average Expression
Percent Expressed
b
CD3E
CD4
CCR7
GPR183
GZMA
CD8A
TRGV9
TNFAIP3
PRF1
FCGR3A
Naive CD4+T
EM/EMRA CD4+T
Naive CD8+T
EM/EMRA CD8+T
gd T
Stressed T
NK1
NK2
c
LYZ
CD14
IL1B
S100A8
S100A12
FCGR3A
CD1C
LILRA4
IL1B+CM
S100A+CM
IM
NM
Unclassified
cDC
pDC
d
MS4A1
IGHD
CD27
CD83
MZB1
Naive B
Memory B
CD83+ B
Plasma B
e
0M
1M
6M
T/NK
Myeloid
B
Platelet
f
Stressed T
NK2
EM/EMRA CD8+T
gd T
EM/EMRA CD4+T
Naive CD4+T
NK1
Naive CD8+T
UMAP1
UMAP2
g
Naïve CD4+ T
EM/EMRA CD4+ T
Naïve CD8+ T
EM/EMRA CD8+ T
gd T
Stressed T
NK1
NK2
Mepo+Resli
Dupilumab
Frequency (%)
Treatmemt duration (month)
h
Naïve B
Memory B
CD83+ B
Plasma B
i
Treatment duration (month)

Supplementary Fig. 2. Dot plot of markers for annotating subtypes of CD4$^+$ T cells

Dot plot displaying the average expression and percent expressed of corresponding markers across subtypes of CD4$^+$ T cells. S0 and S1 represent State 0 and 1, respectively.

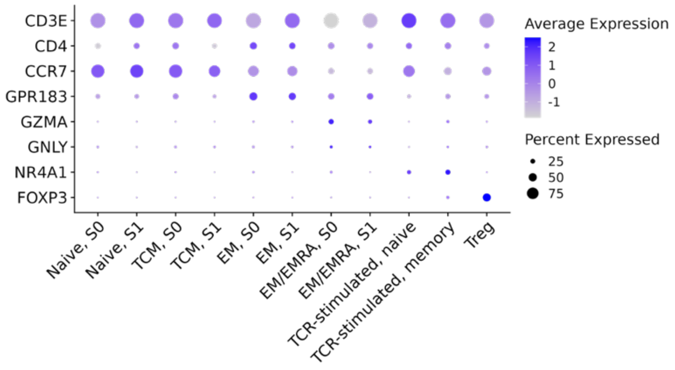

Supplementary Fig. 3. Downregulation of genes involved in the NF-κB pathway in T/NK, myeloid, and B cells

(a) Heatmap displaying the expression levels of DEGs that are significantly different between 6M and 0/1M of T/NK (left panel), myeloid (middle panel), and B cells (right panel) categorized by treatment and duration. The top 20 downregulated and upregulated DEGs in 6M cells compared with 0/1M cells are shown on the y-axis. (b) The top 5 MSigDB Hallmark 2020 pathways and their p-values associated with downregulated DEGs at 6M of T/NK (left panel), myeloid (middle panel), and B cells (right panel). The y-axis represents the names of the pathways, and the x-axis represents -log10 of their p-values. Each pathway has two bars representing the p-values calculated by upregulated DEGs at 0/1M (red) and 6M (turquoise). (c) Top 5 TRRUST transcription factors and their p-values associated with downregulated DEGs at 6M (red colored) of T/NK (left panel), myeloid (middle panel), and B cells (right panel). The turquoise bars represent p-values of the corresponding pathways calculated by upregulated DEGs at 6M.

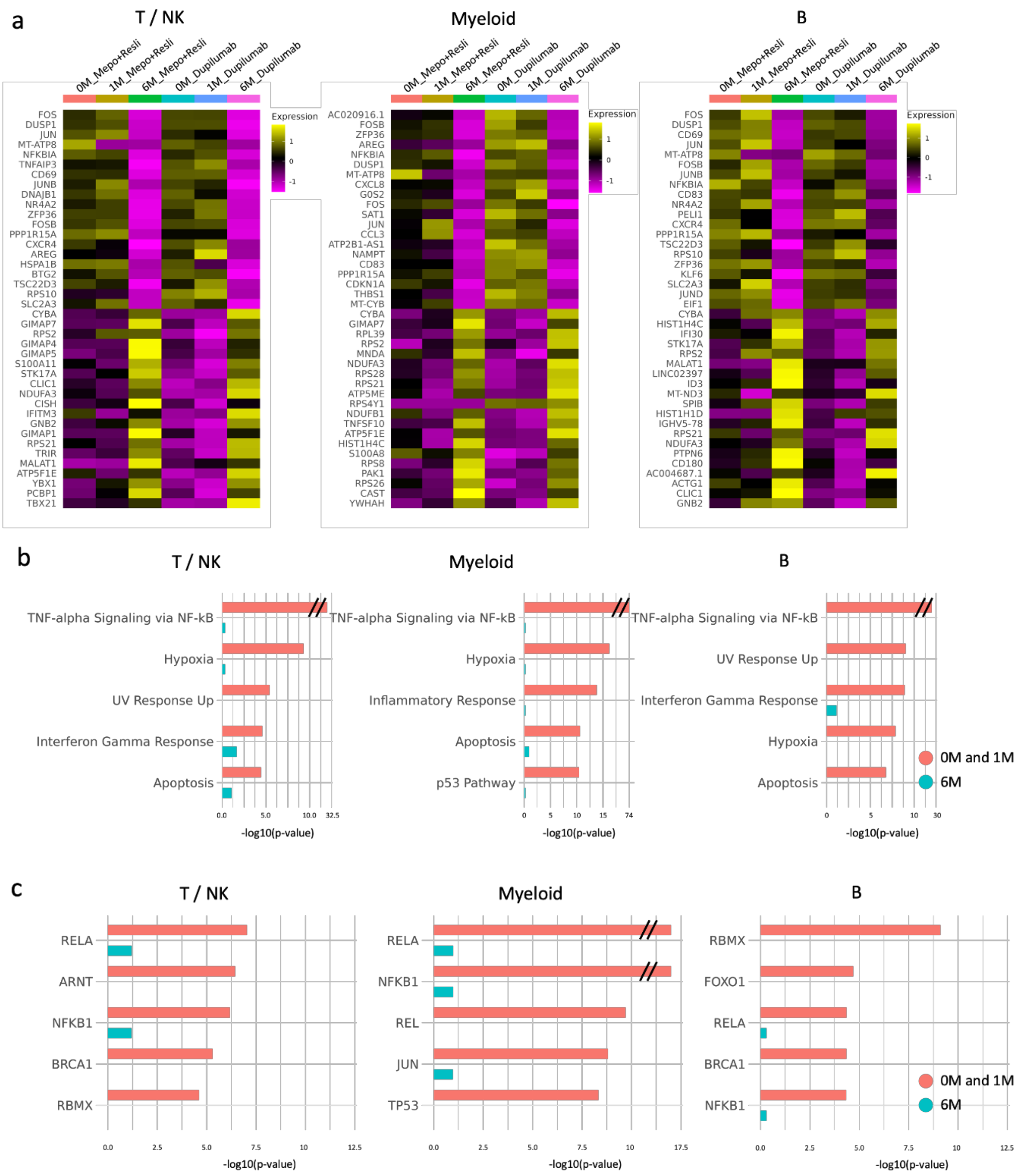
a
T / NK
Myeloid
B
0M_Mepo+Resli
1M_Mepo+Resli
6M_Mepo+Resli
0M_Dupilumab
1M_Dupilumab
6M_Dupilumab
Expression
1
0
-1
FOS DUSP1 JUN MT-ATP8 NFKBIA TNFAIP3 CD69 JUNB DNAJB1 NR4A2 ZFP36 FOSB PPP1R15A CXCR4 AREG HSPA1B BTG2 TSC22D3 RPS10 SLC2A3 CYBA GIMAP7 RPS2 GIMAP4 GIMAP5 S100A11 STK17A CLIC1 NDUFA3 CISH IFITM3 GNB2 GIMAP1 RPS21 TRIR MALAT1 ATP5F1E YBX1 PCBP1 TBX21
AC020916.1 FOSB ZFP36 AREG NFKBIA DUSP1 MT-ATP8 CXCL8 G0S2 FOS SAT1 JUN CCL3 ATP2B1-AS1 NAMPT CD83 PPP1R15A CDKN1A THBS1 MT-CYB CYBA GIMAP7 RPL39 RPS2 MNDA NDUFA3 RPS28 RPS21 ATP5ME RPS4Y1 NDUFB1 TNFSF10 ATP5F1E HIST1H4C S100A8 RPS8 PAK1 RPS26 CAST YWHAH
FOS DUSP1 CD69 JUN MT-ATP8 FOSB JUNB NFKBIA CD83 NR4A2 PELI1 CXCR4 PPP1R15A TSC22D3 RPS10 ZFP36 KLF6 SLC2A3 JUND EIF1 CYBA HIST1H4C IFI30 STK17A RPS2 MALAT1 LINC02397 ID3 MT-ND3 SPIB HIST1H1D IGHV5-78 RPS21 NDUFA3 PTPN6 CD180 AC004687.1 ACTG1 CLIC1 GNB2
b
T / NK
TNF-alpha Signaling via NF-kB
Hypoxia
UV Response Up
Interferon Gamma Response
Apoptosis
0.0 5.0 10.0 32.5
-log10(p-value)
Myeloid
TNF-alpha Signaling via NF-kB
Hypoxia
Inflammatory Response
Apoptosis
p53 Pathway
0 5 10 15 74
-log10(p-value)
B
TNF-alpha Signaling via NF-kB
UV Response Up
Interferon Gamma Response
Hypoxia
Apoptosis
0 5 10 30
-log10(p-value)
0M and 1M
6M
c
T / NK
RELA
ARNT
NFKB1
BRCA1
RBMX
0.0 2.5 5.0 7.5 10 12.5
-log10(p-value)
Myeloid
RELA
NFKB1
REL
JUN
TP53
0.0 2.5 5.0 7.5 10 17.5
-log10(p-value)
B
RBMX
FOXO1
RELA
BRCA1
NFKB1
0.0 2.5 5.0 7.5 10 12.5
-log10(p-value)
0M and 1M
6M

Supplementary Fig. 4. Effect of biologics on communication of CD4⁺ T cells with other cell types based on their mechanisms of action

(a) Raster plots representing the differences in the number of signaling pathway networks among cell types between 6M and 0/1M according to all biologics (left panel), the mepolizumab/reslizumab group (middle panel), and the dupilumab group (right panel). The x-axis and y-axis represent cell types as receptors and ligands, respectively, in a signaling pathway network. The differences are colored ranging from blue (+14 connections) to red (-14 connections). Blue color represents an increase in the number of networks at 6M, and red color represents a decrease in the number of networks at 6M compared with that at 0/1M. (b) Chord plots of MHC-II signaling pathway network at 0/1M and 6M of all patients. (c) Chord plots of the CD40 signaling pathway network at 0/1M and 6M of all patients. S0 and S1 represent State 0 and 1, respectively.

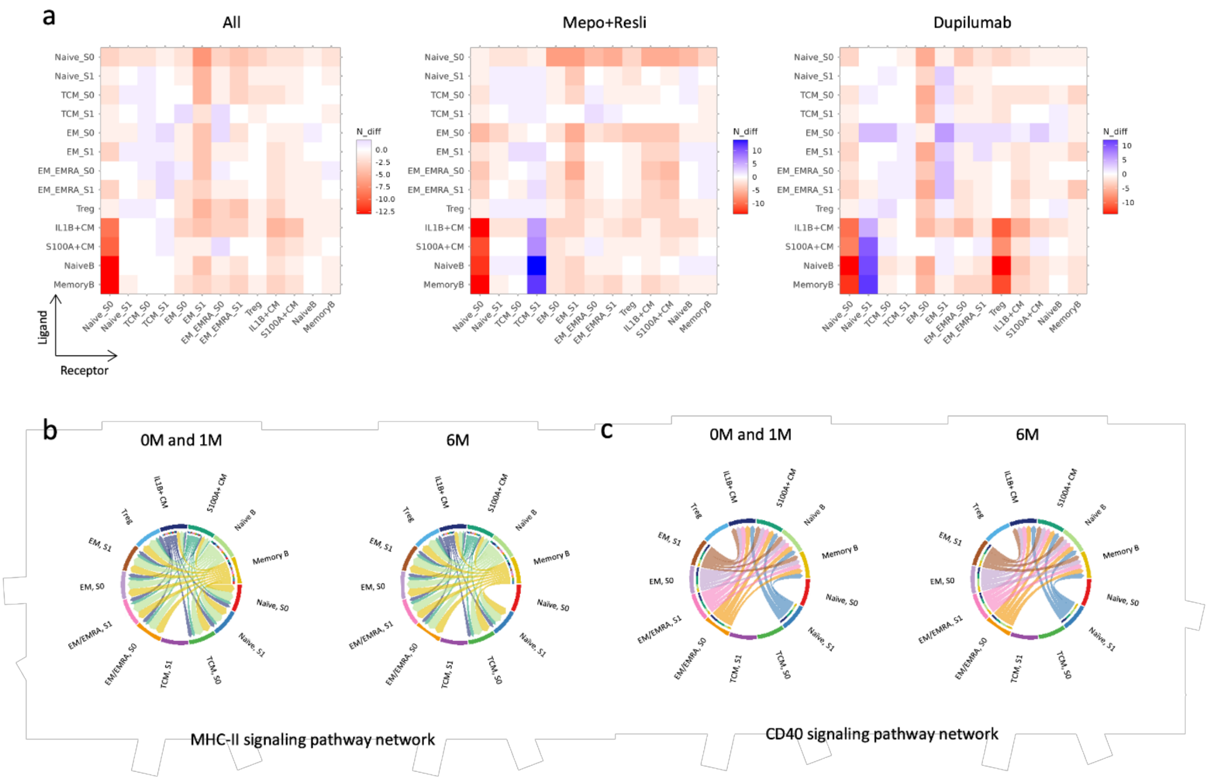

Supplement: Supplementary file 1 — Supplementary materials [file 12276_2024_1368_MOESM1_ESM.pdf]
